# A simulation model to predict agricultural tractor-semi-trailer combination traction performance under different operating conditions

**DOI:** 10.1038/s41598-026-47522-6

**Published:** 2026-04-21

**Authors:** Tarek Fouda, Rashad Hegazy, Kareem Alhamshary

**Affiliations:** 1 Agricultural Engineering Department, Faculty of Agriculture, Tanta University, Tanta, Egypt; 2 Agricultural Engineering Department, Faculty of Agriculture, Kafr Elsheikh University, Kafr El-Sheikh, Egypt

**Keywords:** Modeling, Semi-trailer, Tractor, Transportation, Traction, Rolling-resistance, Draft, Fuel cost, Engineering, Environmental sciences

## Abstract

A computational model was developed to evaluate the performance and design parameters of a tractor–semi-trailer combination operating under different soil conditions. The model predicts key operational parameters including draft force, drawbar power, tractive efficiency, and fuel consumption, while also generating geometric and structural design parameters for the semi-trailer such as loading box dimensions, axle load distribution, suspension configuration, and weight transfer to the tractor drawbar. Soil–tire interaction was represented using traction and rolling resistance relationships derived from established agricultural traction equations. The model was implemented in a Python-based graphical user interface that allows users to evaluate tractor–semi-trailer performance for different operating conditions including soil strength, wheel slip, forward speed, loading capacity, tractor drivetrain configuration, and tire type. Soil conditions were represented using cone index values ranging from 450 to 1800 kPa corresponding to soft sandy, tilled, firm, and hard soil. Model validation was conducted using standardized tractor performance data obtained from official tractor test reports for four agricultural tractors: John Deere 7810, John Deere 7930, New Holland G190, and New Holland TS120. Simulations were performed under identical operating conditions (12% slip and 6 km h⁻^1^ forward speed) to evaluate model scalability across tractors with different power ratings and geometric configurations. Predicted drawbar power requirements ranged from approximately 31 to 105 HP depending on soil strength and tractor characteristics. Comparison with standardized tractor test data showed that predicted power demand represented between approximately 62% and 74% of available drawbar power for most tractors under firm soil conditions. Statistical evaluation indicated stable model performance with a coefficient of variation of approximately 9.7% and a correlation coefficient of R^2 ^≈ 0.84 between predicted and reference drawbar power values. The results demonstrate that the developed model provides realistic predictions of tractor–semi-trailer performance and can be used as a decision-support tool for evaluating transport operations and assisting in the design and selection of tractor–trailer combinations under varying soil and loading conditions.

Transportation represents a critical component of agricultural production systems, particularly in large-scale farming operations where efficiency in time and energy use directly affects productivity and operating cost. Modern agricultural projects increasingly rely on rapid material movement under harsh field conditions, making transportation performance a determining factor in overall farm efficiency.

In Egypt, agricultural mechanization is expanding to support national land reclamation pro-grams, and machinery investment reflects this growth. For example, imports of agricultural machinery reached 17.76 thousand units worth 193.99 million USD in 2022^1^. In parallel, domestic production of agricultural trailers and semi-trailers increased to 824 units in 2020^2^, while the cultivated area reached 55.44 thousand kilometers in 2021 with 133.5 thousand tractors in operation^3^. Transportation operations consume a substantial share of agricultural energy. In Western Europe, transport tasks account for 30–40% of tractor operating time, and similar patterns are emerging in developing countries as mechanization expands^4^.

Agricultural trailers serve diverse applications and weight ranges, and semi-trailers are gaining importance in large desert-based agricultural projects in Egypt. Semi-trailers can transfer part of their load to the tractor, improving stability and maneuverability on irregular terrain, where rocky and uneven soil conditions are common. This configuration also supports higher transport speeds over longer distances, aligning with the requirements of modern large-scale field operations.

Despite increasing adoption, local semi-trailer production capacity remains limited. Historically, the agricultural machinery industry in Egypt has relied on small workshops, and efforts to formalize the sector have been intermittent^5^. The early introduction of imported agricultural semi-trailers inspired initial domestic attempts; however, comprehensive design, performance evaluation, and engineering tools tailored to local soil conditions, tractor characteristics, and load demands are still lacking.

Previous research has contributed valuable digital and analytical tools for agricultural machinery performance assessment. Mohamed et al.^6^ introduced an Excel-based interactive system designed to assist in selecting tractors and field implements, structuring its approach around field capacity calculations and matching power demand with available machinery configurations. Aboukarima^7^ employed a C-Sharp application supplemented by neural-network-derived parameters to support tractor–chisel plow matching and predict operating performance under different field conditions.

Kumar and Pandey^8^ applied Visual Basic to generate an interface that identifies optimal tractor gear and throttle combinations for tillage operations, basing its method on fuel-use models and measured tractor operating behavior. Rocca et al.^9^ used the SEMoLa (Simulation, Easy Modeling Language) simulation platform to construct X-farm, a dynamic modeling framework integrating soil, crop, livestock, and energy modules to support whole-farm decision making across economic and environmental dimensions. Mohamed et al.^10^ used Visual Basic to build a machinery-performance database application that enabled users to input tractor and implement parameters and simulate operating performance using embedded engineering models.

Abbaspour-Gilandeh et al.^11^ similarly used Visual Basic to create a graphical interface for predicting tractor fuel consumption across soil types and tire configurations, embedding traction and propulsion models into an accessible software tool. Al-Hamed and Al-Janobi^12^ developed a Visual C+  + program to predict tractor traction behavior in agricultural soils, incorporating tire databases and traction equations to evaluate performance under different drive-train configurations.

In parallel, studies on tractor–trailer dynamics have emphasized the engineering importance of weight transfer. Li et al.^13^ examined semi-trailer tongue-weight effects on vehicle stability through controlled vehicle tests, characterizing how load distribution influences directional control and safety. Macmillan^14^ and Alcock^15^ discussed optimal static and dynamic weight balance for two- and four-wheel-drive agricultural tractors, grounding the analysis in traction mechanics and steering requirements. Previous studies have demonstrated the usefulness of numerical modelling and simulation tools for analyzing engineering systems and optimizing component performance. For instance, Mohamad et al.^16^ and Mohamad et al.^17^ employed a simulation-based approaches to investigate airflow dynamics and performance characteristics in internal combustion engine intake and exhaust systems, enabling designers to evaluate design alternatives prior to prototype development. Numerical modelling approaches allow engineers to predict stress distribution, deformation, and load-induced responses in vehicle structures prior to physical prototyping. For example, according to Agarwal and Mthembu^18^ finite-element-based simulation and design optimization techniques have been widely applied to heavy-vehicle chassis systems to evaluate stress concentration regions and identify sensitive design parameters affecting structural performance and weight reduction. In articulated vehicle configurations such as tractor–semi-trailer combinations, the dynamic interaction between the tractor unit and the trailer introduces additional complexity, including load transfer, articulation dynamics, and stability issues. In Marumo et al.^19^ High-fidelity simulation frameworks are therefore commonly used to analyze phenomena such as trailer swing, jack-knifing, and stability limits under different loading and operating scenarios.

These efforts collectively demonstrate the importance of traction modeling, load distribution, and computational decision support in agricultural machinery engineering. Existing simulation tools for heavy vehicle analysis primarily focus on detailed multi-body vehicle dynamics or structural finite-element modelling of individual components. Such tools typically require extensive geometric input data and are often designed for highway transport vehicles or high-fidelity dynamic simulations. In contrast, the model proposed in this study is developed specifically for agricultural tractor–semi-trailer applications and focuses on evaluating operational performance under varying loading and field operating conditions. The proposed approach adopts established mechanical and vehicle-dynamics principles for load distribution and traction analysis, while introducing a simplified computational framework capable of estimating drawbar forces, load transfer, and operational performance indicators relevant to agricultural transport systems.

Despite the depth of research in tractor performance modeling and machinery selection tools, there remains a clear gap in engineering frameworks focused on agricultural semi-trailer design, power demand, and tractor–trailer load interaction under the soil and operating conditions typical of Egyptian large-scale farming systems. The theoretical basis of the model adopts established formulations from classical vehicle dynamics and traction mechanics, including load equilibrium, drawbar force analysis, and resistance modelling. However, several aspects are newly implemented in this work. These include the integration of tractor power characteristics with trailer loading conditions within a unified simulation framework, the implementation of parametric analysis for varying payload configurations, and the development of a computational procedure capable of evaluating tractor–semi-trailer operational performance under agricultural transport conditions.

Accordingly, this study aims to develop a semi-trailer design and performance evaluation model tailored to agricultural operations, quantify draft force and power requirements, and ex-amine the interaction between semi-trailer, tractor, and soil parameters.

## Materials and methods

A computational model was developed to predict the performance of a tractor–semi-trailer combination under different operating conditions. The model calculates draft force, drawbar power, tractive efficiency, and fuel consumption based on tractor specifications, semi-trailer design parameters, soil conditions, and operating variables.

Microsoft Excel® was used to implement the mathematical model and perform the required calculations. The spreadsheet contains all model inputs, intermediate parameters, equations, and outputs associated with the prediction of draft force and power requirements for agricultural semi-trailers. The simulations were executed on an HP Z420 workstation equipped with an Intel® Xeon® CPU E5-1650 (3.20 GHz), 16 GB RAM, NVIDIA Quadro M2000 graphics card, and running Windows 10 Pro (64-bit). The graphical user interface (GUI) was developed using Python IDLE 3.13.4.

### Excel mathematical model development

The Excel® model consists of three interconnected worksheets. The first worksheet, Design Data, stores all constants, coefficients, and reference data required for the calculations. These data are organized in tabular format and linked to the input and output cells to ensure dynamic interaction between parameters.

The second worksheet, Semi-Trailer Design, contains the primary model inputs and corresponding outputs. Each parameter is organized in a separate row with a unique identifier to facilitate tracking of relationships between variables. All dependent parameters are automatically updated when the user modifies any input value. The third worksheet, Results, summarizes the calculated performance parameters and illustrates the effects of design variables and operating conditions.

The model operates through four computational stages: Input stage, where the user provides soil, tractor, and trailer parameters. Measurement stage, including tractor and semi-trailer geometric dimensions. Intermediate calculations, where derived parameters are computed. Output stage, where the model predicts performance indicators such as draft force, power requirement, and tractive efficiency.

**Fig. 1:**
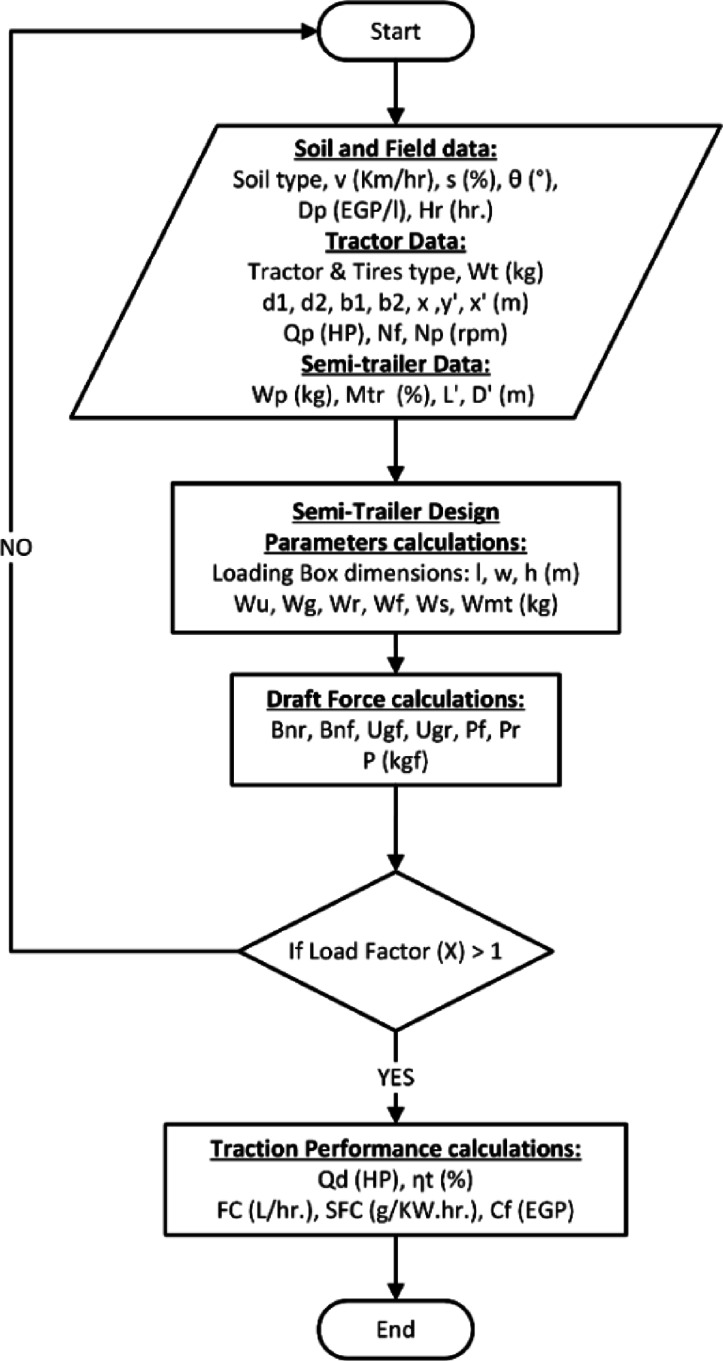
Flowchart of semi-trailer tractor combination performance prediction.

A schematic representation of the computational workflow is shown in (Fig. 1), which illustrates the sequence of calculations used to evaluate the performance of the tractor–semi-trailer combination.

### Model inputs

The required model inputs include soil conditions, tractor specifications, operating parameters, and semi-trailer loading characteristics.

Soil conditions are selected through a drop-down menu in the Excel interface. Four soil types are available, each associated with a corresponding cone index (CI, kPa) adopted from ASAE standards^20^. The selected value is automatically integrated into the model calculations.

The user also specifies the following operating parameters: wheel slip, $s$(%), travel speed, $v$(km h⁻^1^), field slope direction (no slope, uphill, or downhill) and slope angle, $\theta$(°).

Tractor configuration is defined by selecting either a two-wheel drive or four-wheel drive tractor. The weight distribution ratio on the drive axle ${M}_{r}$ is automatically assigned based on the selected tractor type. For two-wheel drive tractors, the rear and front axle weight distribution ratios are 0.7 and 0.3 respectively, while for four-wheel drive tractors they are 0.6 and 0.4, as reported by Alcock^15^.

Additional tractor parameters include rated PTO power ${Q}_{p}$(HP), full-throttle engine speed ${N}_{f}$(rpm), partial-throttle engine speed ${N}_{p}$(rpm), diesel fuel price ${D}_{p}$(EGP L⁻^1^) and operating time ${H}_{r}$(h).

Tractor tire characteristics are also specified, including tire type (bias-ply or radial-ply), tire diameters ${d}_{1}$, ${d}_{2}$, and section widths ${b}_{1}$, ${b}_{2}$(m).

The transported material is selected from a predefined list of agricultural materials. Each material is associated with a density value $\rho$(ton m⁻^3^), which is automatically applied in the model. The semi-trailer payload ${W}_{p}$(kg) is then specified by the user.

The volumetric capacity of the trailer is calculated as

1 $$V = \frac{{W_{p} }}{\rho }$$

where: $V$: semi-trailer volumetric capacity (m^3^), ${W}_{p}$: semi-trailer payload (kg), $\rho$: loading material density (kg m⁻^3^).

### Semi-trailer loading box dimensions calculation

Based on the calculated volumetric capacity, the dimensions of the loading box are determined.

The loading box length $L$(m) is assigned according to the payload capacity: ${W}_{p}\ge 18$ t (L = 6.7 m), ${W}_{p}\ge 10$ t (L = 6 m), ${W}_{p}\ge 5$ t (L = 5 m) or ${W}_{p}<5$ t (L = 4 m).

Similarly, loading box width $W$(m) is defined as: ${W}_{p}\ge 10$ t (W = 2.5 m) or $5\le {W}_{p}<10$ t (W = 2 m).

The loading box height $H$(m) is calculated as:

2 $$H = \frac{V}{W \times L}$$

Drawbar length $D$(m) is determined according to PAES 136^21^, which specifies that the distance from the foremost end of the loading platform to the hitch point should range between 0.9 and 1.4 m.

### Semi-trailer load calculation

The unladen mass of the semi-trailer is assumed to be 40% of the payload capacity:

3 $$W_{u} = 0.4W_{p}$$

where: $W_{u}$ is the unladen mass (kg).

The gross trailer load is then calculated as

4 $$W_{g} = W_{p} + W_{u}$$

The weight transfer ratio from the semi-trailer to the tractor drawbar ${M}_{tr}$ is limited to 20% of the gross trailer weight according to IS 8213^22^. The transferred weight is therefore

5 $$W_{mt} = M_{tr} \times W_{g}$$

The suspension load supported by the trailer axles is

6 $$W_{s} = W_{g} - W_{mt}$$

Based on this load, the axle configuration is selected automatically: ${W}_{s}\ge 18$ t (tridem axle), ${W}_{s}\ge 12$ t (tandem axle) or ${W}_{s}<12$ t (single axle). The axle load ${W}_{a}$ is then determined accordingly.

### Tractor semi-trailer combination weight transfer parameters

Geometric parameters of the tractor and semi-trailer are required to determine the distribution of forces within the system. These parameters include tractor mass ${W}_{t}$, wheelbase $x$, drawbar height $y^{\prime }$, and horizontal distance from the rear axle to the drawbar $x^{\prime }$. Semi-trailer parameters include loading box length $L^{\prime }$ and drawbar length $D^{\prime }$.

The suspension spacing from the rear end of the loading box $G$ is determined according to PAES 136^21^ and IS 8213^22^:

7 $$G = \frac{{\left( {W_{g} L)/2 - W_{mt} (L + D} \right)}}{{W_{g} - W_{mt} }}$$

The distance between the suspension center and the tractor drawbar is

8 $$a = \left( {L + D} \right) - G$$

**Fig. 2:**
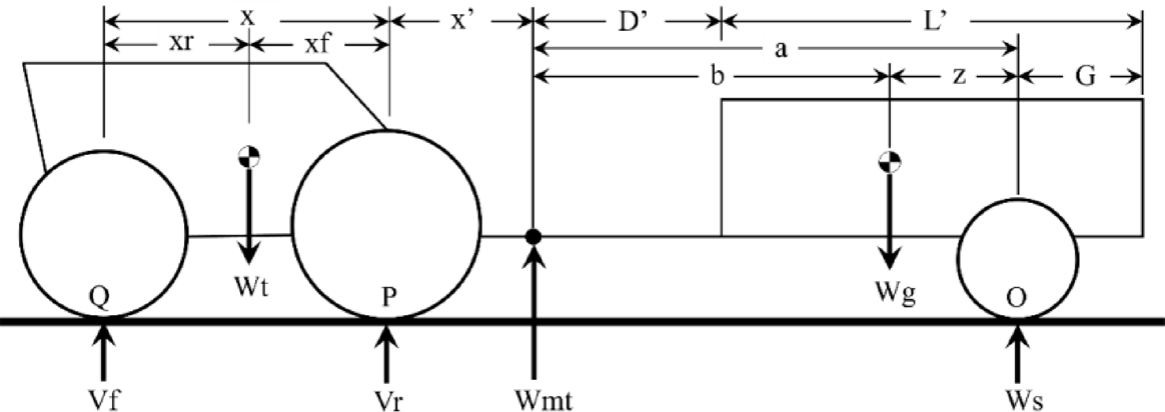
Tractor semi-trailer combination free body diagram.

The horizontal position of the semi-trailer center of gravity is obtained from moment equilibrium (Fig. 2):

$$z = \frac{{W_{mt} a}}{{W_{g} }}$$

The distance between the center of gravity and the towing eye is

9 $$b = a - z$$

The total static weight of the tractor–trailer combination is

10 $$W_{tr} = W_{t} + W_{mt}$$

The static loads on the tractor rear and front axles are

11 $$W_{r} = M_{r} W_{tr}$$

12 $$W_{f} = W_{tr} - W_{r}$$

### Tractor tire and soil conditions parameters

In the developed model, this interaction is represented through empirical traction relationships that relate wheel slip, soil strength, and normal wheel load to traction and rolling resistance coefficients. These relationships are widely used in agricultural tractor performance analysis and are derived from classical terra-mechanics principles describing the shear behavior of soil under tire loading. The model incorporates soil mechanical conditions through the soil cone index parameter, which influences both traction and rolling resistance coefficients. Changes in wheel slip modify the available traction force generated at the tire–soil interface, while the normal load acting on the tractor wheels is influenced by weight transfer from the semi-trailer drawbar. Through these parameters, the model captures the primary mechanisms governing soil–tire interaction during tractor transport operations.

Tire and soil properties are combined in the mobility number ${B}_{n}$, calculated according to Goering et al.^23^.

Rear tire mobility number:

13 $$B_{nr} = \frac{{CI\,b_{r} d_{r} }}{{W_{r} }}\left( {\frac{{1 + 5\delta_{r} /h_{r} }}{{1 + 3b_{r} /d_{r} }}} \right)$$

Front tire mobility number:

14 $$B_{nf} = \frac{{CI\,b_{f} d_{f} }}{{W_{f} }}\left( {\frac{{1 + 5\delta_{f} /h_{f} }}{{1 + 3b_{f} /d_{f} }}} \right)$$

A typical tire deflection ratio $\delta /h=0.2$ is assumed as recommended by Alcock^15^.

The traction coefficient ${\mu }_{g}$ and rolling resistance coefficient $\rho$ are calculated using the empirical relationships proposed by Goering et al.^23^ for bias-ply tires as:

15 $$\mu_{g} = 0.88\left( {1 - e^{{ - 0.1B_{n} }} } \right)\left( {1 - e^{ - 7.5s} } \right) + 0.04$$

16 $$\rho = \frac{1}{{B_{n} }} + 0.04 + \frac{0.5s}{{\sqrt {B_{n} } }}$$

And for radial-ply tires as:

17 $$\mu_{g} = 0.88\left( {1 - e^{{ - 0.1B_{n} }} } \right)\left( {1 - e^{ - 9.5s} } \right) + 0.0325$$

18 $$\rho = \frac{0.9}{{B_{n} }} + 0.0325 + \frac{0.5s}{{\sqrt {B_{n} } }}$$

Traction and rolling resistance coefficients were calculated using empirical traction relationships rather than treated as calibration parameters. These coefficients vary dynamically as a function of wheel slip, soil cone index, and wheel load.

### Tractor semi-trailer combination traction parameters

The drawbar pull $P$ is determined from the traction force generated at the rear axle minus the rolling resistance at the front axle, as described by Macmillan^14^:

19 $$P=\mu {V}_{r}-\rho {V}_{f}$$

Dynamic axle loads ${V}_{r}$ and ${V}_{f}$ are obtained from static equilibrium analysis using the free-body diagram shown in (Fig. 2). The resulting expression for draft force is:

20 $$P = \frac{{W_{r} \mu + W_{g} \mu \frac{{\left( {a - b)(x + x^{\prime}} \right)}}{ax} - W_{f} \rho + W_{g} \rho \frac{{x^{\prime}\left( {a - b} \right)}}{ax}}}{{1 - \mu \frac{{y^{\prime}\left( {a + x + x^{\prime}} \right)}}{ax} - \rho \frac{{y^{\prime}\left( {a + x^{\prime}} \right)}}{ax}}}$$

Slope effects are incorporated as:

21 $$P_{uphill} = P + \left( {W_{g} + W_{t} } \right){\mathrm{sin}}\theta$$

22 $$P_{downhill} = P - \left( {W_{g} + W_{t} } \right){\mathrm{sin}}\theta$$

Tractive efficiency is calculated as:

23 $$\eta_{t} = \frac{P}{{V_{r} \left( {\mu_{gr} + \rho_{r} } \right)}}\left( {1 - i} \right)$$

Required drawbar power is:

24 $$Q_{d} = Pv\left( {1 - i} \right)c$$

where $c=0.00134$ converts watts to horsepower.

### Fuel consumption calculation

The calculated drawbar power is converted to equivalent PTO power using ASAE standards^20^. The load factor is:

25 $$X = \frac{{Q_{p} }}{{Q_{r} }}$$

The partial throttle multiplier is:

26 $$PTM = 1 - \left( {N_{p} /N_{f} - 1} \right)\left( {0.45X - 0.877} \right)$$

Specific fuel consumption volume is:

27 $$SFC_{v} = \left( {0.22 + 0.096/X} \right)PTM$$

Fuel consumption rate is:

28 $$FC = SFC_{v} Q_{d}$$

An additional 15% fuel consumption is added to account for field operating losses according to ASAE^24^. Total fuel cost is:

29 $$C_{f} = FC \times D_{p} \times H_{r}$$

### Python desktop GUI development

To facilitate practical use of the model, a desktop application was developed in Python using the Tkinter graphical user interface framework. AI-assisted development tool ChatGPT by OpenAI Company has been used for scripting support and troubleshooting during the coding process. The application includes four functional tabs: Inputs, Dimensions, Outputs, and About.

The Inputs tab allows the user to enter tractor specifications, soil conditions, and semi-trailer design parameters. Drop-down menus are provided for parameters such as soil type, tractor configuration, tire type and loading material. Users can modify or add new entries through an integrated editing interface.

The Dimensions tab displays the tractor–semi-trailer free-body diagram (Fig. 2) to assist users in entering the required geometric parameters. The Outputs tab presents the calculated design and performance results, including draft force, tractive efficiency, required power, and fuel consumption.

#### Model evaluation scenarios

To clarify the simulation design, the ranges of parameters used in the model evaluation are summarized in (Table 1).

**Table 1:** Simulation parameters and ranges used in model evaluation.

Parameters	Range	Step/cases
Soil cone index (kPa)	450–1800	4 soil types
Slip (%)	2–40	2% increment
Forward speed (km h⁻^1^)	2–40	5 speeds
Trailer load (kg)	5000–24,000	5 load levels
Tractor type	2WD, 4WD	2 types
Tire type	Bias, Radial	2 types

A sensitivity analysis was conducted to evaluate the robustness of the model predictions under varying operational conditions. The parameter ranges used in the simulation were selected to represent typical operating conditions encountered in agricultural transport operations. Soil cone index values ranging from 450 to 1800 kPa correspond to soft sandy soils, tilled soils, firm cultivated soils, and compacted field surfaces commonly encountered in agricultural fields according to ASAE traction data^20^. Wheel slip was varied from 2 to 40%, covering both normal tractor operating conditions and extreme slip levels. In practical agricultural operations, the optimal slip range for efficient traction typically lies between 8 and 15%, while higher slip values represent inefficient operating conditions. Forward travel speeds from 2 to 40 km h⁻^1^ represent the practical operating range of agricultural tractors during transport operations, including low-speed field movement and higher-speed transport on farm roads.

In addition, the trailer load was varied from 5 to 24 t to examine the influence of load variation on traction performance. Although the nominal loading capacity of the developed semi-trailer is 16 t, higher loads were considered in the simulation in order to evaluate model sensitivity near the upper operational limits of the tractor–semi-trailer system. These parameter selections allow the developed model to represent a wide range of realistic tractor–semi-trailer operating conditions and therefore support its applicability to agricultural transport systems beyond the specific baseline configuration used in this study. The effects of these parameter variations on draft force, drawbar power, tractive efficiency, and fuel consumption are presented and discussed in the Results section.

#### Model validation using tractor test reports

The developed tractor–semi-trailer performance model was validated using standardized tractor performance data obtained from official tractor test reports^25–28^. Four agricultural tractors representing different power classes and geometric configurations were selected for evaluation: John Deere 7810, John Deere 7930, New Holland G190, and New Holland TS120. The maximum drawbar power values reported in the tractor test reports were used as reference performance indicators.

To ensure a consistent comparison, all tractors were simulated under identical operating conditions. The forward speed was fixed at 6 km h⁻^1^ and the wheel slip was maintained at 12%, corresponding to the typical optimal slip range for agricultural traction performance. Additional operating parameters were kept constant, including drivetrain configuration (4WD), tire type (bias-ply), semi-trailer loading capacity (12,000 kg), transported material (sugar beet), and weight transfer ratio (15%). Tractor-specific parameters such as mass, wheelbase, tire dimensions, drawbar height, and rated PTO power were entered individually for each tractor in compatible with the tractor test reports.

Model predictions of required drawbar power were compared with the maximum drawbar power values reported in the tractor test reports. Several quantitative indicators were used to evaluate model behavior, including power utilization ratio, mean absolute percentage error (MAPE), coefficient of variation (CV), and coefficient of determination (R^2^). Validation indicators calculated as:

30 $$Power Utilization\left( \% \right) = \frac{Predicted drawbar power}{{Maximum drawbar power}} \times 100\left( {30} \right)$$

31 $$MAPE = \frac{1}{n} \sum \left( {\frac{{\left| {Predicted - Measured} \right| }}{Measured}} \right) \times 100\left( {31} \right)$$

32 $$CV = \frac{Standard deviation}{{Mean}} \times 100$$

33 $$R^{2} = 1 - \frac{{\Sigma \left( {Measured - Predicted} \right)^{2} }}{{\Sigma \left( {Measured - Mean measured} \right)^{2} }}$$

## Results

Several simulation scenarios were conducted using the developed model in order to evaluate the effects of different operating conditions and design parameters on the performance of the tractor–semi-trailer combination. A dedicated worksheet named “Results” was created in the model to apply various parameter combinations and visualize their effects on the predicted performance variables.

Model predictions presented in this section represent simulation outputs generated using the developed computational model. These results are subsequently compared with available experimental field data to evaluate the predictive capability of the model.

The baseline conditions used in all simulated cases were kept constant except for the parameter under investigation. The initial conditions were as follows: soil condition firm, loading material sugar beet, tractor forward speed 10 km h⁻^1^, wheel slip 10%, and no field slope. The tractor model considered was John Deere 7810, configured as a four-wheel drive tractor equipped with bias-ply tires. Front and rear tire sizes were 14.9R28 and 18.4R38, respectively. The John Deere 7810 tractor was selected as the baseline configuration because it represents a widely used medium-power agricultural tractor commonly employed for trailer transport operations under Egyptian field conditions. In addition, this tractor was used during the field test of the locally developed semi-trailer, as shown in (Fig. 8). The rated PTO power was 166 HP, with full throttle speed 2100 rpm and partial throttle speed 1400 rpm.

The semi-trailer loading capacity was 12,000 kg, with a 15% weight transfer ratio from the semi-trailer to the tractor. The diesel fuel price was 17.5 EGP L⁻^1^, and the daily operating period was 8 h.

The outputs generated by the developed model can be classified into two categories: performance parameters and design parameters. The performance parameters describe the operational behavior of the tractor–semi-trailer combination under different operating conditions. (Table 2) summarizes the predicted tractor–semi-trailer performance parameters under the baseline operating conditions.

**Table 2:** Predicted tractor–semi-trailer performance parameters under baseline conditions.

Parameter	Value
Draft force (kgf)	3460
Drawbar power (HP)	113.74
Tractive efficiency (%)	59.12
Fuel consumption (L h⁻^1^)	26.87
Specific fuel consumption (g kW⁻^1^ h⁻^1^)	234.43

In addition to predicting traction performance, the developed model generates geometric and structural design parameters for the semi-trailer based on the selected loading capacity and material density. (Table 3) summarizes the main design parameters obtained under the same baseline operating conditions.

**Table 3:** Predicted semi-trailer design parameters generated by the model.

Parameter	Predicted value
Maximum loading box length (m)	6.7
Maximum loading box width (m)	2.5
Maximum loading box height (m)	0.895
Distance from suspension center to tail (m)	2.124
Weight transferred to tractor drawbar (kg)	2520
Gross trailer load (kg)	16,800
Suspension load (kg)	14,280
Suspension configuration	Tandem
Axle load (kg)	7140

Both the traction performance results and the predicted semi-trailer design parameters can be visualized in the developed Python graphical user interface, as shown in (Fig. 4).

The calculated suspension configuration for the baseline scenario was a tandem axle system, with an axle load within typical design limits for agricultural transport trailers. This configuration is consistent with the design used in the locally developed semi-trailer, as shown in (Fig. 5). These results demonstrate that the developed model can support both performance prediction and preliminary semi-trailer design.

### Field and operational parameters affecting semi-trailer performance

#### Soil type effect on traction performance

The influence of soil conditions on tractor–semi-trailer traction performance was analyzed while maintaining all other parameters constant. Soil conditions were varied by modifying the soil cone index (CI) corresponding to different soil types.

The tested soil types, according to ASAE^20^ data included hard, firm, tilled, and soft (sandy) soils with cone index values of 1800, 1200, 900, and 450 kPa, respectively.

The results indicated that decreasing the soil cone index resulted in a decrease in traction coefficient, draft force, required drawbar power, tractive efficiency, and fuel consumption rate and an increase in rolling resistance coefficient and specific fuel consumption.

These results demonstrate that softer soils reduce traction capability, which consequently affects the energy efficiency of the tractor–semi-trailer system.

#### Slip effect on traction performance

**Fig. 3:**
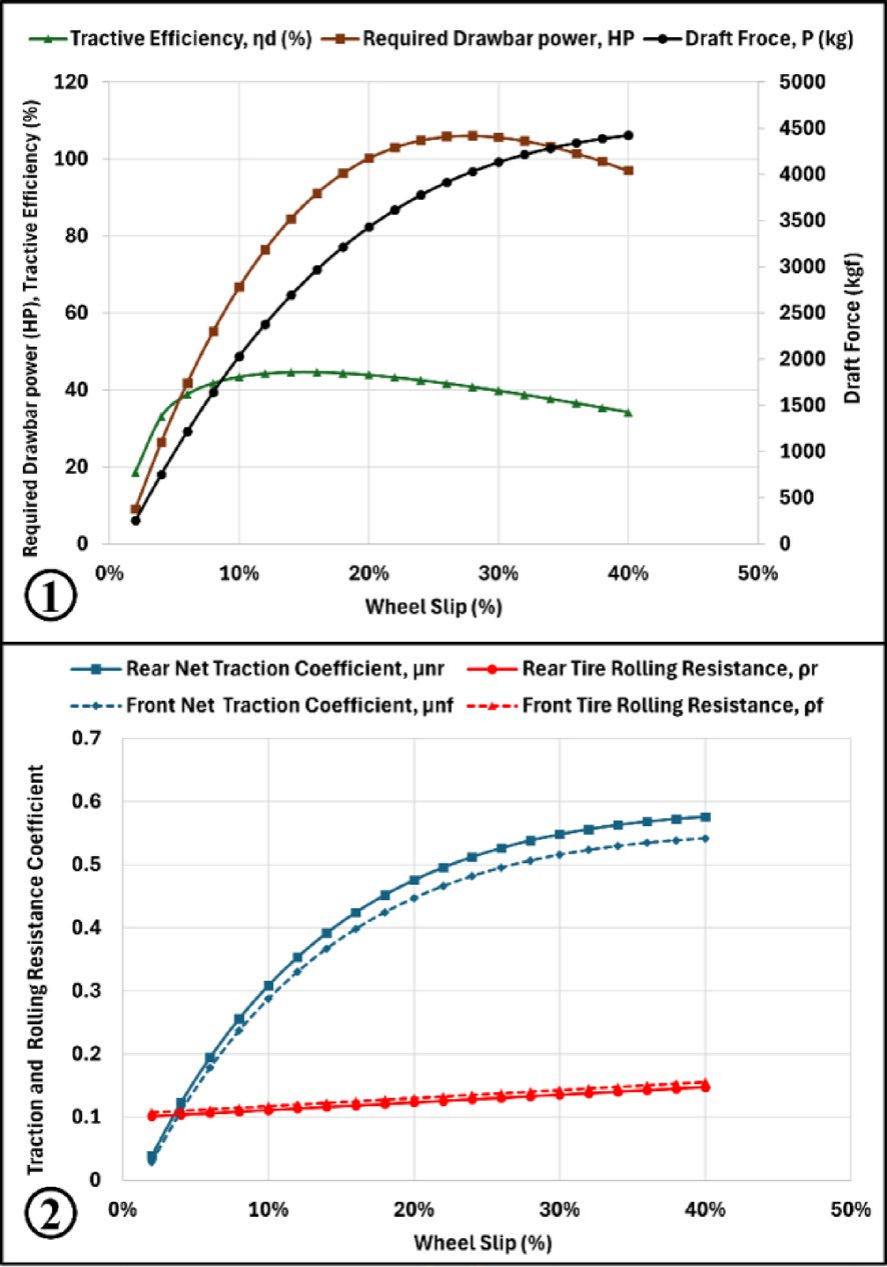
Predicted behavior of draft force, drawbar power and tractive efficiency (1), traction and rolling resistance coefficients (2) against wheel slip variation.

Wheel slip was varied from 2 to 40% while maintaining a constant travel speed of 10 km h⁻^1^. The results revealed a direct relationship between slip and draft force. Required drawbar power increased with increasing slip until approximately 30% slip, after which it began to decline, as shown in (Fig. 3). A similar trend was observed for tractive efficiency, which increased with slip until reaching a maximum at approximately 20% slip, and then gradually decreased. Both traction coefficient and rolling resistance coefficient increased with increasing slip.

Fuel consumption increased with slip up to approximately 25% slip, after which it began to decrease. Conversely, specific fuel consumption decreased as slip increased up to 25%, followed by a slight increase at higher slip values. These findings indicate the existence of an optimum slip range where traction efficiency and power utilization are maximized.

#### Tractor type effect on traction performance

The influence of tractor drive configuration was examined by comparing two-wheel drive (2WD) and four-wheel drive (4WD) tractors. In this scenario, tractor forward speed was varied from 4 to 40 km h⁻^1^, and wheel slip was varied from 4 to 40%, while all other parameters remained constant.

The results showed that 4WD tractors required lower draft force for operating the semi-trailer compared with 2WD tractors. Consequently, the required drawbar power was also lower for the 4WD configuration. Although 2WD tractors exhibited slightly higher tractive efficiency, they required greater draft force and higher power input than 4WD tractors.

In addition, 4WD tractors showed higher traction coefficients and lower rolling resistance coefficients compared with 2WD tractors. Fuel consumption and fuel cost were also higher for 2WD tractors, particularly at travel speeds exceeding 15 km h⁻^1^. However, the specific fuel consumption per unit power was similar for both tractor configurations. Overall, the results indicate that 4WD tractors provide more favorable traction performance and lower fuel consumption when operating semi-trailers under the examined conditions.

#### Tractor tire type effect on traction performance

The influence of tire construction was evaluated by comparing radial-ply tires and bias-ply tires. In this case, forward speed was varied from 4 to 40 km h⁻^1^, and slip ranged from 4 to 40%, while all other parameters remained unchanged. The results showed that radial-ply tires required slightly higher draft force compared with bias-ply tires, resulting in higher required drawbar power. However, radial-ply tires produced higher traction coefficients and lower rolling resistance coefficients, leading to improved tractive efficiency.

The maximum tractive efficiency for radial-ply tires occurred at forward speeds between 5 and 15 km h⁻^1^, whereas bias-ply tires achieved their maximum efficiency within the 10 to 20 km h⁻^1^ speed range. Although radial-ply tires demonstrated better traction efficiency, they resulted in higher fuel consumption and operating cost compared with bias-ply tires. Specific fuel consumption, however, remained similar for both tire types, particularly within the 10–20 km h⁻^1^ speed range.

#### Loading capacity effect on traction performance

Semi-trailer loading capacity was varied from 5000 to 24,000 kg while keeping all other parameters constant. Increasing the loading capacity resulted in higher draft force, greater weight transfer from the trailer to the tractor, increased rolling resistance coefficient, higher required drawbar power and increased fuel consumption and fuel cost.

**Fig. 4:**
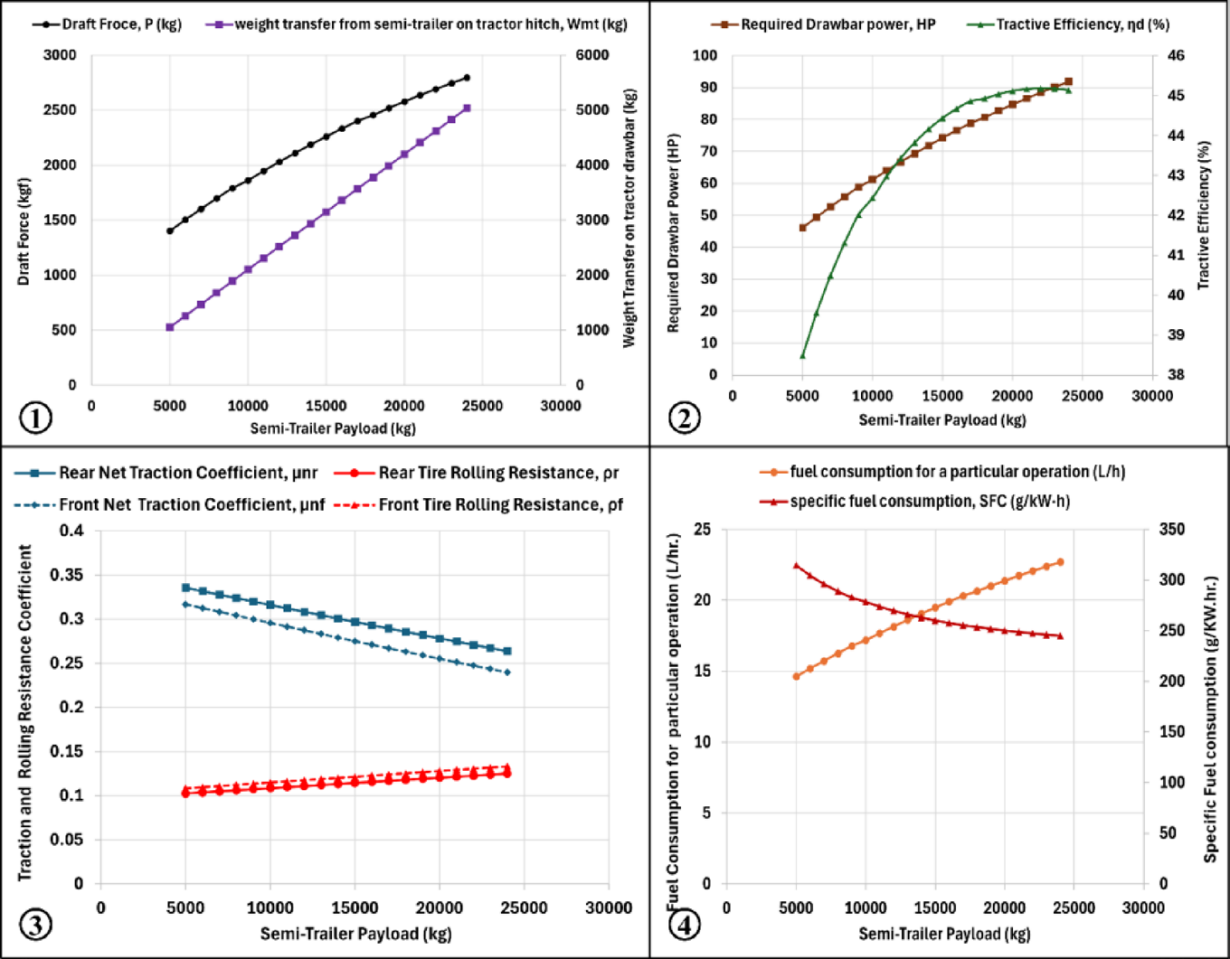
Predicted behavior of draft force and weight transfer (1), drawbar power and traction efficiency (2), traction and rolling resistance coefficients (3), fuel consumption and specific fuel consumption (5) against wheel semi-trailer payload increasing.

Conversely, increasing the loading capacity caused a decrease in traction coefficient, tractive efficiency, and specific fuel consumption, as shown in (Fig. 4). These results highlight the strong influence of trailer load on the overall energy requirements of tractor–trailer systems.

### Combined effects of slip and forward speed under different soil conditions

Unlike the previous cases where only one parameter was varied, this analysis simultaneously examined the effects of forward speed and wheel slip under four different soil conditions. Forward speed was varied from 5 to 25 km h⁻^1^, and slip ranged from 5 to 25%, while other parameters remained constant.

**Fig. 5:**
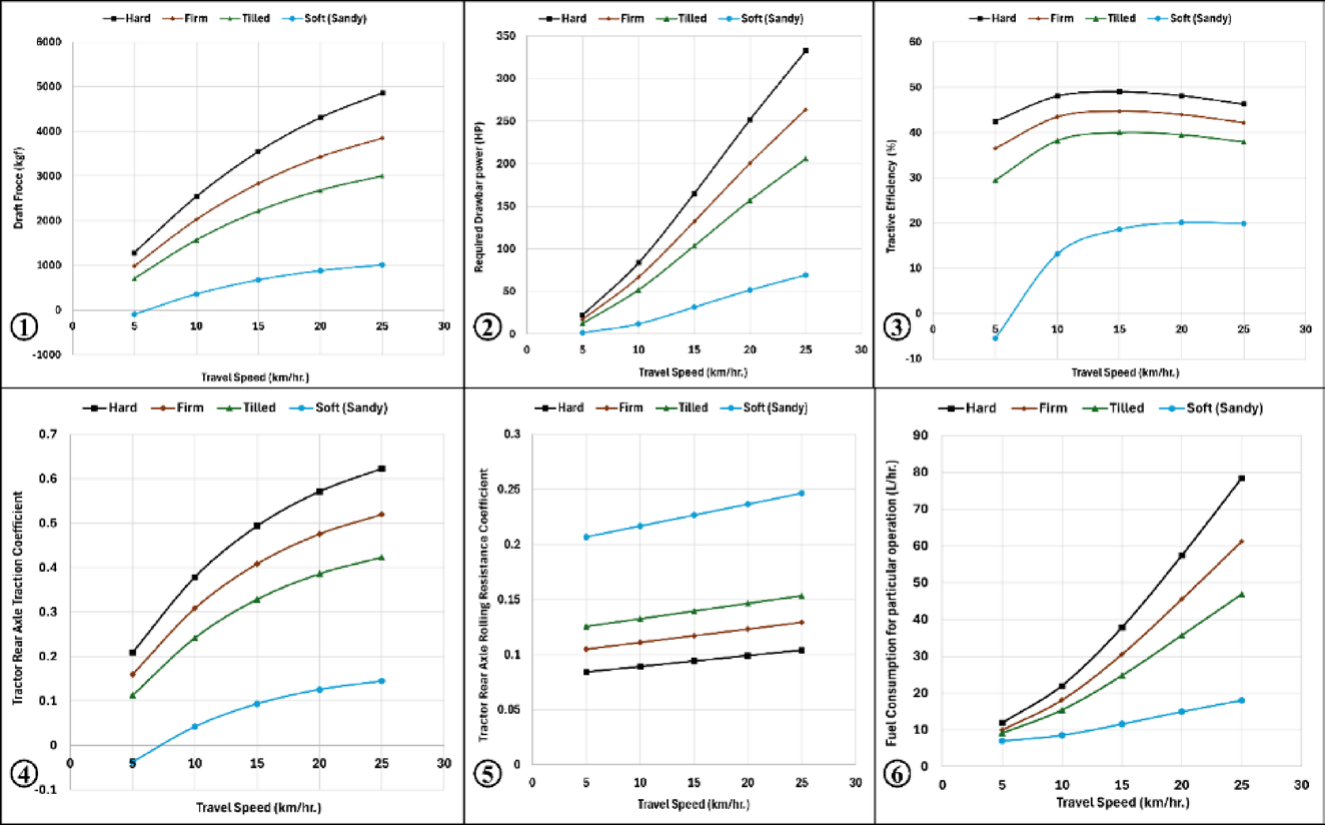
Traction performance and fuel consumption prediction for Tractor Semi-trailer combination for different soils and travel speeds: (1) draft force, (2) drawbar power, (3) tractive efficiency, (4) traction coefficient, (5) rolling resistance coefficient and (6) fuel consumption.

As illustrated in (Fig. 5), increasing both forward speed and slip resulted in higher draft force and required drawbar power across all soil types. Similarly, both traction coefficient and rolling resistance coefficient increased with increasing speed and slip. Harder soils with higher cone index values resulted in higher fuel consumption and operating costs, whereas specific fuel consumption decreased with increasing soil strength.

### Slip effects for different forward speeds

**Fig. 6:**
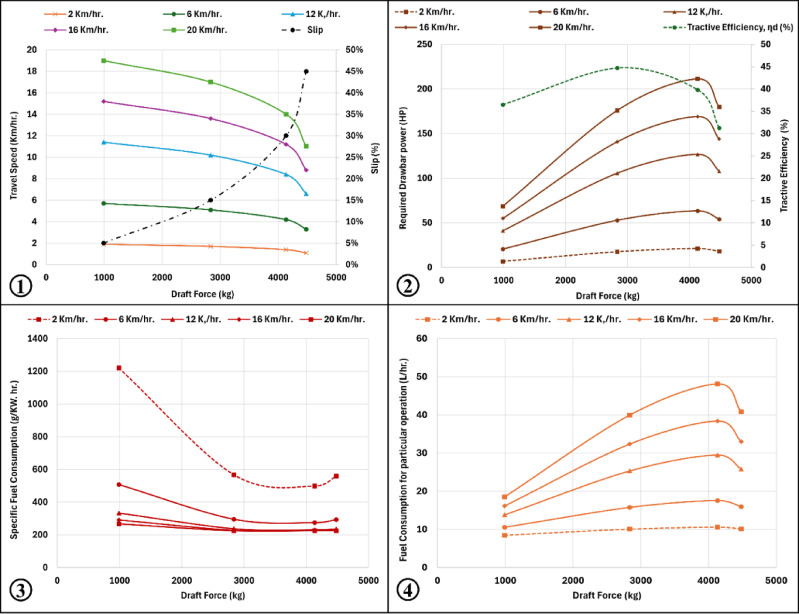
Traction performance and fuel consumption prediction for Tractor Semi-trailer combination based on available draft force: (1) slip and travel speed, (2) drawbar power and traction efficiency, (3) specific fuel consumption and (4) fuel consumption.

In this case, wheel slip was varied from 2 to 32% while maintaining all other conditions constant. The analysis was repeated for five forward speeds: 2, 6, 12, 16, and 20 km h⁻^1^. The traction parameters were plotted against draft force P (kgf) to better illustrate the interaction between slip and draft force. As shown in (Fig. 6), draft force increased with increasing slip, while tractor operating speed decreased as draft force increased. Required drawbar power increased with slip until reaching a maximum at approximately 30% slip, after which it began to decline. Tractive efficiency showed a similar pattern, reaching its maximum at approximately 15% slip before decreasing at higher slip levels. Specific fuel consumption decreased within the 5–30% slip range but began to increase when slip exceeded 30%. Conversely, fuel consumption and fuel cost increased up to 30% slip, then gradually declined at higher slip levels.

### Python GUI desktop application

The developed Python-based GUI application was used to predict the performance of a 16-ton tipping semi-trailer operated behind a John Deere 7810 tractor during sugar beet harvesting operations.

In the Inputs tab, the soil condition closest to the actual field was selected as firm, and approximate values of travel speed and slip were entered based on field observations. The loading material was selected as beet, and the weight transfer ratio was set to 15%. Tractor and semi-trailer geometric parameters were entered according to manufacturer specifications.

**Fig. 7:**
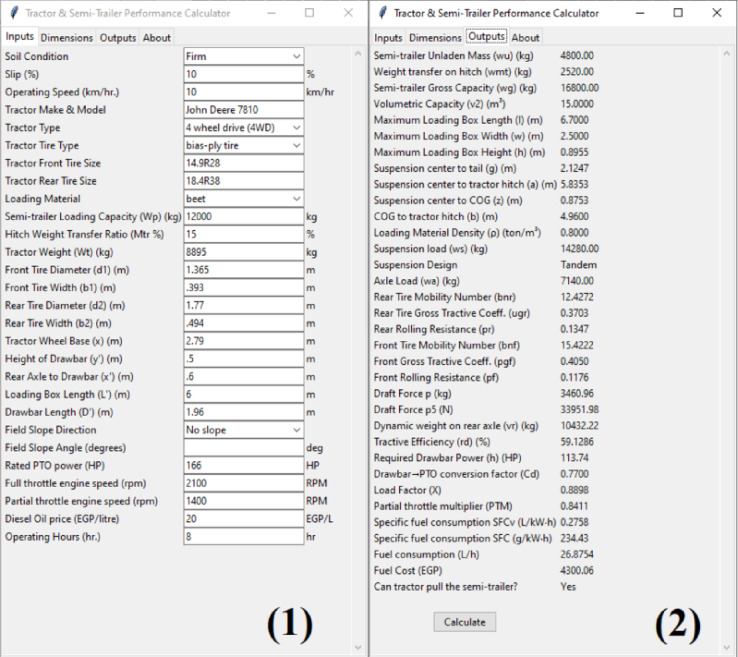
Tractor semi-trailer combination performance GUI desktop application (1) Inputs tab, (2) Outputs tab.

The Outputs tab displayed the calculated semi-trailer load distribution, loading box geometry, and tractor–semi-trailer traction parameters. The predicted performance values were Draft force: 3460 kgf, Required drawbar power: 113.74 HP, Tractive efficiency: 59.12%, Specific fuel consumption: 234.43 g kW⁻^1^ h⁻^1^, Fuel consumption rate: 26.87 L h⁻^1^ and Fuel cost: 4300 EGP. (Fig. 7) shows the graphical interface of the developed application, including the Inputs and Outputs tabs.

**Fig. 8:**
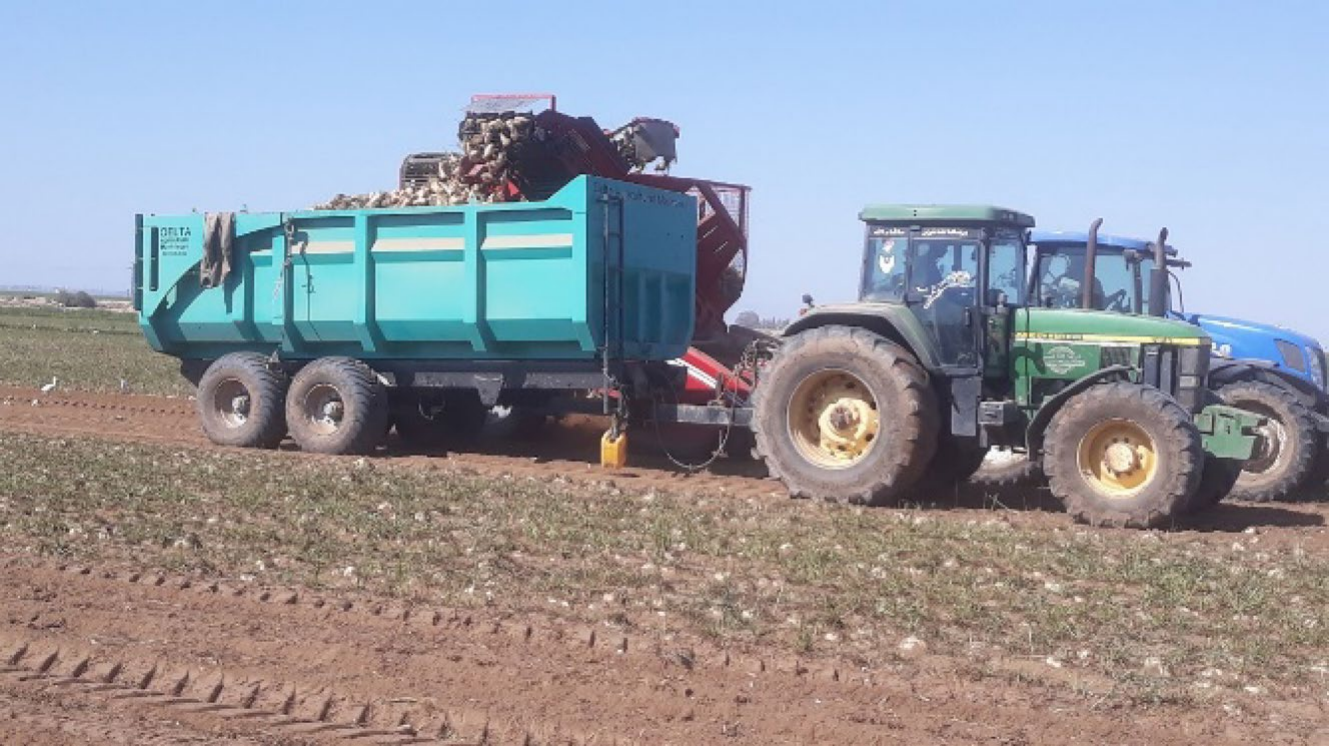
John Deere 7810 Tractor and Local developed Semi-trailer combination during sugar beet harvest, Ismailia Governorate, Near Tell El-kebir (2023).

The predicted drawbar power fell within the manufacturer’s specified drawbar power range for the tractor and was also consistent with the results reported in John Deere tractor test report No. 1807^25^. This agreement indicates that the developed model is capable of reliably predicting the drawbar power requirements of the tractor–semi-trailer combination.

(Fig. 8) presents the field operation of the John Deere 7810 tractor coupled with the locally developed semi-trailer during the sugar beet harvest season in Ismailia Governorate near Tell El-Kebir (2023).

### Model validation

To provide quantitative validation of the model, the predicted drawbar power was compared with the value reported in the tractor test reports^25–28^. The predicted drawbar power obtained for all tractors in different soil conditions are listed in (Table 4). The predicted drawbar power requirements for the tractor–semi-trailer combination were compared with the maximum drawbar power values reported in standardized tractor test reports. The results indicated that the predicted power demand varied systematically with both tractor power rating and soil strength. For hard soil conditions, the predicted drawbar power ranged between 83.8 and 105.5 HP depending on tractor configuration. As soil strength decreased from hard soil to soft sandy soil, the predicted drawbar power requirement decreased accordingly due to the reduced traction capability of loose soils.

**Table 4:** Predicted drawbar power for different soil conditions generated by the model.

Tractor	Max drawbar power (HP)	Predicted drawbar power (HP)			
		Hard soil	Firm soil	Tilled soil	Soft soil
John Deere 7810	139.47	91.6	75.5	62.4	31.18
John Deere 7930	159.10	98.65	82.4	68.7	35.14
New Holland G190	142.17	105.5	87.9	73.2	37.94
New Holland TS120	87.99	83.8	71.38	60.62	34.38

The predicted power requirements remained within the operational capability of the tested tractors. Power utilization ranged from approximately 62% to 74% of available drawbar power for three tractors under hard soil conditions, while the smaller tractor reached approximately 95% utilization. Statistical indicators showed stable model behavior with a coefficient of variation of approximately 9.7% across tractors under hard soil conditions. The overall mean absolute percentage error between predicted and reference drawbar power values was approximately 25.7%, while the coefficient of determination between predicted and measured values reached approximately R^2^ = 0.84.

Direct field measurements of tractive efficiency and fuel consumption were not available during the field experiment. However, the predicted values fall within the typical ranges reported in agricultural tractor performance studies according to ASAE standards^20,22^ and the tractor test reports.

## Discussion

Soil mechanical properties strongly influenced the predicted behavior of the tractor–semi-trailer system. As expected, soils with higher cone index values exhibited higher traction coefficients and lower rolling resistance compared with loose soils. This behavior reflects the greater load-bearing capacity of cohesive soils, which allows tractor tires to transmit larger shear forces without excessive deformation of the soil surface. Consequently, cohesive soils required higher draft force and drawbar power to move the semi-trailer but simultaneously provided better tractive efficiency than loose soils. These findings are consistent with classical traction theory and previously reported observations for agricultural tractors operating under different soil strengths. The predicted drawbar power requirement decreased as soil strength decreased due to the reduced traction capability of loose soils. Hard soils provided greater shear resistance to the tractor tires, allowing higher tractive forces and therefore larger draft forces to be transmitted to the semi-trailer. In contrast, soft sandy soils limited the achievable traction force because soil failure occurred at lower stress levels, resulting in lower draft and drawbar power requirements.

Wheel slip also showed a strong influence on traction performance. Increasing slip increased the draft force required to pull the semi-trailer because higher slip corresponds to greater shear deformation at the tire–soil interface. However, drawbar power and tractive efficiency increased only within a limited slip range and decreased at higher slip levels. The results indicated that a slip range of approximately 10–20% produced the highest tractive efficiency, which agrees with values commonly recommended for maximizing tractive efficiency in agricultural tractor operations. Similar traction behavior has been described by Macmillan^14^ and Alcock^15^, who showed that moderate slip levels allow efficient conversion of engine power into useful drawbar work while excessive slip results mainly in energy losses.

The tractor drive configuration also affected the predicted performance of the tractor–semi-trailer system. Although two-wheel-drive tractors showed slightly higher tractive efficiency values, four-wheel-drive tractors required lower draft force and drawbar power to operate the semi-trailer under the same conditions. The improved performance of four-wheel-drive tractors is primarily related to the more balanced weight distribution and the contribution of both axles to traction generation, which reduces the load carried by individual tires and improves soil–tire interaction. As a result, four-wheel-drive tractors also showed lower fuel consumption compared with two-wheel-drive tractors, particularly at higher travel speeds.

Tire construction type further influenced traction performance. Radial-ply tires produced higher traction coefficients and lower rolling resistance coefficients than bias-ply tires, resulting in improved tractive efficiency. This behavior is consistent with the greater flexibility of radial tire sidewalls, which allows a larger contact area with the soil surface. However, the simulations indicated that radial-ply tires required slightly higher draft force and drawbar power compared with bias-ply tires under the studied conditions. Although radial tires provided improved traction efficiency, they also resulted in higher fuel consumption during operation.

Trailer loading capacity had a direct influence on the traction requirements of the tractor–semi-trailer combination. Increasing the payload increased draft force, rolling resistance, required drawbar power, and fuel consumption. This behavior is physically expected because the additional mass increases the vertical load acting on the tractor–trailer system and therefore increases the resistance forces that must be overcome during transport. At the same time, the model predicted a decrease in traction coefficient and tractive efficiency with increasing trailer load, indicating that higher loads reduce the efficiency of power transfer between the tractor and the ground.

The combined analysis of soil type, slip, and travel speed further illustrated the interaction between soil mechanical properties and tractor operating conditions. Cohesive soils generally required higher draft force and drawbar power but also allowed higher traction coefficients compared with loose soils. The simulations indicated that tractive efficiency was lowest at travel speeds below approximately 10 km h⁻^1^ and slip values below 10%, whereas higher efficiencies were obtained within moderate ranges of speed and slip. For cohesive soils, the optimal operating region was approximately 10–15 km h⁻^1^ travel speed and 10–15% slip, while loose soils required slightly higher speeds and slip levels to achieve comparable performance.

The results also indicated that increases in traction coefficient in cohesive soils were accompanied by only minor increases in rolling resistance, suggesting favorable traction conditions where sufficient thrust can be generated with relatively low resistance. In contrast, loose soils showed nearly proportional increases in traction and rolling resistance coefficients, indicating that resistance forces may rapidly approach the available traction force under soft soil conditions. This behavior was particularly evident for sandy soils, where rolling resistance approached or exceeded the available traction coefficient, making semi-trailer operation more difficult.

Overall, the results demonstrate that the developed model can provide useful predictions of both traction performance and semi-trailer design parameters under a wide range of operating conditions. By combining soil characteristics, tractor specifications, and trailer design variables, the model can support decision making for selecting suitable operating conditions and trailer configurations in agricultural transport operations. Also, The developed Python-based graphical user interface (GUI) was designed with a modular structure that allows users to modify input parameters such as tractor power, soil cone index, operating speed, and trailer loading capacity. This structure enables the model to be applied to a wide range of tractor–semi-trailer configurations without modifying the underlying computational framework. Therefore, the application can be extended to evaluate different tractor power classes and trailer designs commonly used in agricultural transport operations.

The validation results demonstrate that the developed model responds realistically to variations in tractor power rating and soil strength. Higher cone index soils provided greater shear resistance to the tractor tires, allowing higher traction forces to be transmitted to the ground and resulting in increased drawbar power requirements. Conversely, soft sandy soils limited the achievable traction force due to early soil failure, which reduced the usable drawbar power despite higher rolling resistance coefficients.

The predicted power utilization values fall within typical ranges reported for agricultural transport operations, where tractors commonly operate between 40 and 80% of their available drawbar power depending on soil and loading conditions. The relatively low coefficient of variation across tractors indicates stable model behavior and suggests that the simulation framework responds consistently to changes in tractor design parameters such as mass, tire size, and wheelbase. The strong correlation between predicted and measured drawbar power further supports the validity of the model as a decision-support tool for evaluating tractor–semi-trailer performance under varying soil conditions.

### Model limitations

The developed model is based on empirical traction relationships and assumes uniform soil conditions and steady-state tractor operation. Field variability such as soil heterogeneity, transient slip conditions, operator behavior, operation and maintenance conditions are not explicitly represented in the model. As a result, prediction accuracy may decrease under highly variable soil moisture conditions or extreme field slopes. The model assumes steady-state tractor operation and therefore does not explicitly simulate transient dynamic loading effects caused by terrain irregularities, acceleration, or vibration. In practical field conditions, these dynamic effects may cause short-term variations in wheel slip and drawbar load. However, for typical agricultural transport operations conducted at relatively constant speeds, steady-state traction relationships provide a reasonable approximation of tractor–semi-trailer performance.

In addition, model validation was conducted using a single tractor model and one field test scenario. Although the predicted drawbar power showed good agreement with the available tractor test data, further validation using additional tractor models, soil conditions, and field experiments would improve the robustness and general applicability of the model.

## Conclusion

A computational model was developed to predict the traction performance and design parameters of a tractor–semi-trailer transport system under different soil and operating conditions. The model estimates draft force, drawbar power, tractive efficiency, fuel consumption, and traction characteristics, while also generating key semi-trailer design parameters such as loading box dimensions, axle load, suspension configuration, and drawbar weight transfer.

The simulation results demonstrated that soil mechanical properties significantly influence tractor–semi-trailer performance. Cohesive soils generally produced higher traction coefficients and lower rolling resistance than loose soils, resulting in improved tractive efficiency, although higher draft force and drawbar power were required to move the trailer. Soil strength also influenced fuel consumption, with harder soils generally requiring greater engine power during operation.

Operating parameters such as wheel slip and forward speed were found to strongly affect traction behavior. Moderate slip values in the range of approximately 10–20% provided the highest tractive efficiency, confirming commonly reported optimal slip ranges for agricultural tractors. Increasing operating speed and slip increased draft force and drawbar power requirements, leading to higher fuel consumption during transport operations.

Trailer loading capacity had a direct effect on traction requirements. Increasing payload increased draft force, rolling resistance, and fuel consumption, while also increasing weight transfer to the tractor drawbar. Excessive loading may therefore reduce overall tractive efficiency and increase the power required for transport operations. Maintaining a weight transfer ratio within the recommended range of approximately 10–15% can help maintain stable tractor–trailer interaction and appropriate load distribution.

The results also indicated that tractor configuration and tire type influence traction performance. Four-wheel-drive tractors generally required lower draft force and drawbar power compared with two-wheel-drive tractors under similar operating conditions, while radial-ply tires provided higher tractive efficiency due to improved soil–tire contact characteristics.

Validation of the developed tractor–semi-trailer performance model was conducted using four tractors with different power ratings and configurations. The predicted drawbar power requirements ranged from approximately 31 HP to 105 HP depending on soil strength and tractor characteristics. Comparison with standardized tractor test data showed that the predicted power demand represented between 62 and 74% of the available drawbar power for most tractors under firm soil conditions. Statistical evaluation yielded a coefficient of variation of approximately 9.7% and a correlation coefficient of R^2 ^≈ 0.84 between predicted and reference drawbar power values. These results indicate that the developed model provides realistic and stable predictions of tractor–semi-trailer performance.

Overall, the developed model can serve as a practical decision-support tool for evaluating tractor–semi-trailer performance and preliminary trailer design under different soil and operating conditions. By combining traction prediction with design parameter estimation, the model can assist operators and designers in selecting appropriate operating conditions, tractor configurations, and trailer dimensions for agricultural transport applications.

## Data Availability

The input parameters used in the simulations, including tractor specifications, soil cone index values, and trailer design parameters, are provided within the manuscript tables. Example simulation dataset used to generate the results are included in the Results section to facilitate reproducibility. The complete Excel model is available from the corresponding author upon reasonable request (Tarek Fouda, tfouda628@gmail.com).
